# Non-intersecting leaf insertion algorithm for tree structure models

**DOI:** 10.1098/rsfs.2017.0045

**Published:** 2018-02-16

**Authors:** Markku Åkerblom, Pasi Raumonen, Eric Casella, Mathias I. Disney, F. Mark Danson, Rachel Gaulton, Lucy A. Schofield, Mikko Kaasalainen

**Affiliations:** 1 Laboratory of Mathematics, Tampere University of Technology, PO Box 553, 33101 Tampere, Finland; 2 Centre for Sustainable Forestry and Climate Change, Forest Research, Farnham GU10 4LH, UK; 3 Department of Geography, University College London, Gower Street, London WC1E 6BT, UK; 4 NERC National Centre for Earth Observation (NCEO), UK; 5 School of Environment and Life Sciences, University of Salford, Salford M5 4WT, UK; 6 School of Engineering, Newcastle University, Newcastle upon Tyne NE1 7RU, UK; 7 School of Humanities, Religion and Philosophy, York St John University, York YO31 7EX, UK

**Keywords:** leaf insertion, leaf distribution, quantitative structure model, laser scanning, tree reconstruction

## Abstract

We present an algorithm and an implementation to insert broadleaves or needleleaves into a quantitative structure model according to an arbitrary distribution, and a data structure to store the required information efficiently. A structure model contains the geometry and branching structure of a tree. The purpose of this work is to offer a tool for making more realistic simulations of tree models with leaves, particularly for tree models developed from terrestrial laser scanning (TLS) measurements. We demonstrate leaf insertion using cylinder-based structure models, but the associated software implementation is written in a way that enables the easy use of other types of structure models. Distributions controlling leaf location, size and angles as well as the shape of individual leaves are user definable, allowing any type of distribution. The leaf generation process consist of two stages, the first of which generates individual leaf geometry following the input distributions, while in the other stage intersections are prevented by carrying out transformations when required. Initial testing was carried out on English oak trees to demonstrate the approach and to assess the required computational resources. Depending on the size and complexity of the tree, leaf generation takes between 6 and 18 min. Various leaf area density distributions were defined, and the resulting leaf covers were compared with manual leaf harvesting measurements. The results are not conclusive, but they show great potential for the method. In the future, if our method is demonstrated to work well for TLS data from multiple tree types, the approach is likely to be very useful for three-dimensional structure and radiative transfer simulation applications, including remote sensing, ecology and forestry, among others.

## 1. Introduction

Leaves and needles are essential for the functioning of plants and their interaction with the environment. They are also the main part of the vegetation interacting with remote sensing measurements. Thus, the ability to measure and model leaf distributions of plants has great importance and many applications in ecology, forest research and remote sensing [1–3].

We will present an algorithm to generate leaf cover on any plant structure model with any underlying distribution for the leaf parameters. Although the process could be used with any type of plant, this article focuses only on trees. The leaf parameter distributions are supported by quantitative structure models (QSMs) of trees, and the generated leaves are non-intersecting. This allows, among other things, the use of more realistic leaf distributions in gap fraction- and radiative transfer-based simulations, in comparison with the previously suggested uniform layers of possibly intersecting leaves [4].

The above-ground biomass of a tree consists mainly of leaves, and woody material in the trunk and branches. In recent years, various methods have been presented to reconstruct the woody parts of a tree in a quantitative manner from terrestrial laser scanning (TLS) data [5,6]. Furthermore, it is possible to estimate foliage distribution from similar data [7] (for further information, see [8]). However, reconstructing both the woody and leaf parts at the same time is more challenging due to self-occlusion effects, and the complex nature of leaf–wood separation from TLS data, which has been studied extensively [9,10].

An alternative to *extracting* the leaves from TLS data is scanning the tree during the leaf-off season, and then trying to *insert* leaves after reconstructing the woody structure. To generate a leaf cover that is statistically similar to the original, certain leaf property distributions have to be estimated [11]. Such approaches do not aim to reconstruct real leaves but rather the underlying leaf distribution, which can be sampled to produce leaf covers that are statistically similar to the real one. The approach is limited to deciduous, broadleaf canopies. However, from this we may learn how to improve and develop methods for separation and re-insertion of green material in evergreen broadleaf and needleleaf trees.

Measuring leaf position, size and orientation by hand is extremely laborious [11] as one can have millions of leaves per tree. Great progress in measurement systems and data analysis has meant that remote sensing can now be used to detect leaf properties. Methods have been presented to estimate the three-dimensional distribution of leaf material from TLS data [7,12]. Furthermore, methods for measuring leaf orientation distribution (LOD) from similar data have been presented in [13] and more recently in [14]. Determining leaf size distribution (LSD) remotely is more challenging as it requires the detection of leaf edges [15], which is also challenging due to the decrease in data point density higher in the canopies, when scanning from the ground. However, sampling leaf size by hand is faster and less error prone than leaf angle, especially when carried out in a destructive manner.

**Figure 1.**
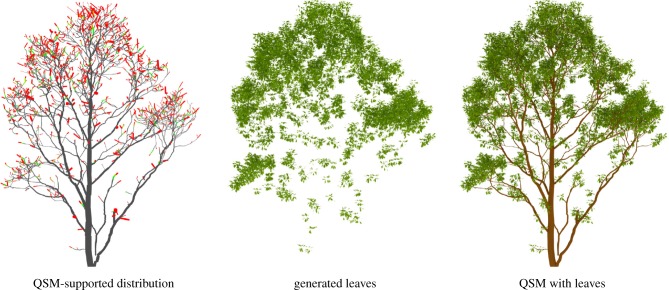
A QSM supports a leaf area distribution (grey: no leaves; green: some leaves; red: a lot of leaves), which can be sampled to generate non-intersecting leaves and inserted into the structure model.

The algorithm we present in this paper populates a QSM of the woody parts of a tree with leaves, resulting in a model with inserted leaves (L-QSM). The algorithm generates leaves based on user-defined leaf property distributions that may be estimated with the methods presented above, or alternatively by using distributions parametrized by branch properties such as branch order. The basic steps of the procedure are illustrated in figure 1, which shows an example leaf area distribution supported by a QSM, leaves generated by sampling the distribution and the final product, which is an L-QSM.

The algorithm is designed to work with models consisting of any type of geometry, but we use models that are a collection of cylinders, i.e. cylindrical QSMs [5]. The leaf insertion procedure works on *blocks*, which is essentially the largest unit of the structure model that can be assumed to have uniform leaf distribution parameters that can define, for example, limits for the number of leaves, leaf size and orientation. Because certain tree species can have a different leaf density along branches, the blocks can be smaller than the branch. Thus, the cylinders forming the QSM geometry, and other similar small geometric primitives [16], can be used directly as blocks. However, it would also be possible to divide the cylinders and form even smaller blocks. In the case of voxel-based structure models a pre-processing step is required to form blocks that are a collection of voxels. Similarly, in continuous surface models the branch surfaces should be divided into smaller sections that can be used as blocks.

As the leaf insertion algorithm is designed to be as general as possible, i.e. any user-defined distribution can be used, validation can take various forms. We carried out initial validation using leaf area and count measurements from three English oaks together with their QSMs reconstructed from TLS data. Both the TLS and leaf measurements are presented in §2.1. The structure reconstruction process to create the required cylindrical QSMs is briefly described in §2.2.

The leaf insertion algorithm is presented in §2.3 together with the related distributions that control leaf position, size and orientation. Although this paper focuses on sampling the described distributions to produce individual leaves with a known geometry, it is not always necessary, as discussed in §2.4. Section 2.4 shows how the distributions define a leaf density distribution around the structure model blocks, and how that overall distribution can be used for computations without generating the geometry of individual leaves. Although we focus on broadleaves, the procedure can also be used for generating needles. Approaches for working with needles are presented in §2.5.

A Matlab implementation of the algorithm, including descriptions of the related classes and the main function, is introduced in §2.6. The Matlab implementation was used to compute several leaf distributions for the oak trees. The results are presented in §3. A discussion is included in §4 and conclusions are made in §5.

## 2. Material and methods

### 2.1. Laser scanning and leaf measurements

Our analysis was based on raw point-clouds recorded at Alice Holt Forest, UK (51.1533 N, 0.8512 W), by a single-return phase-shift Leica HDS-6100 TLS (Leica Geosystems Ltd, Heerbrugg) on three 80-year-old oak trees (*Quercus robur* L.). Scans were performed in March 2014, during winter time, under dry and low wind speed (less than 1 ms^−1^) conditions. Trees were recorded from six scan positions around each tree (azimuth angle of 0° S, 60°, 120°, 180°, 240° and 300°) at a distance of 5 m from the base of the tree and with a TLS sampling resolution level of 0.018° at each scan position. Six reflective targets were set out around each tree to merge the multiple scans. Three-dimensional reconstructions of the trees were then computer-generated using the method described in [5].

The trees were harvested in June 2014. The foliage sampling method consisted of a manual stripping-off of each leaf from the branches and storage in bags labelled with the height stratum to which they belonged (table 1). A second component of the method involved the collection of a set of 100 leaves at random from each stratum on each tree. Each stratum bag was then fresh-weighed (Avery Berkel HL206, UK) and oven dried at 75°C to obtain their dry masses. From the subsets, individual leaf area was measured in the laboratory with a laser area metre (CID-203, Camas, WA, USA) and weighed (Mettler Toledo AG204, Switzerland) before and after oven drying at 75°C. Specific leaf area (SLA) was derived for each of the subsets and used to estimate the total leaf area and the number of leaves for each stratum (e.g. [12,17]). Additionally, the average area of the leaves was recorded from the smallest to the largest tree as 33.71, 40.33 and 29.66 cm^2^, respectively.

**Table 1.** Leaf area and count measurements.

tree/layer	leaf area (m^2^)	leaf count
small oak	153	47 644
0.0–11.5 m	18	5432
11.5–19.6 m	135	42 212
medium oak	215	52 416
0.0–9.0 m	46	12 753
9.0–19.9 m	169	39 663
large oak	339	114 224
0.0–8.0 m	61	16 056
8.0–13.0 m	23	9399
13.0–18.4 m	49	19 597
18.4–22.4 m	206	69 172

### 2.2. Quantitative structure models

**Figure 2.**
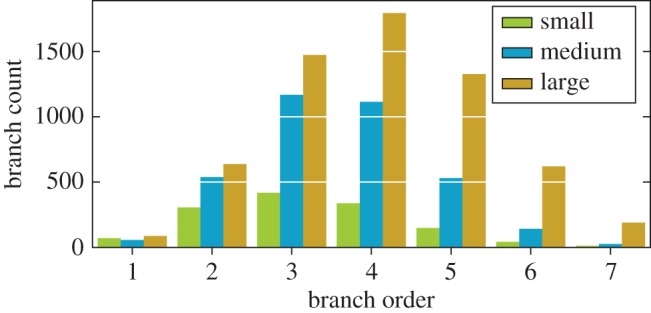
Branch order–count distribution. The stem and branch orders 8 and 9 have been excluded due to their negligible portions. (Online version in colour.)

The three oak trees were reconstructed as cylindrical QSMs in Matlab with the procedure detailed in [18]. The properties of the resulting models are listed in table 2. Furthermore, the branch count distribution per branch order is visualized in figure 2. The count of the branches is important as leaves are placed near the tips of the branches.

**Table 2.** Oak tree properties computed from reconstructed QSMs.

	oak tree		
property	small	medium	large
branch count	1334	3579	6161
cylinder count	8429	23 539	35 428
DBH (mm)	298	432	848
height (m)	19.1	19.6	21.8
order max.	9	8	9
total length (m)	592	1552	2516
volume (l)	707	1169	2098

The small and medium oaks were similar in height, but the latter had about 2.6 times more branches when measured in total count and in length. The large oak had the most branches for all branch orders, and almost twice the volume of the medium oak.

### 2.3. Leaf generation algorithm

This section describes an algorithm to populate QSMs with leaves. The main inputs of the algorithm are distributions that control the position, orientation and size of the leaves. These distributions are sampled to retrieve the parameters of individual leaves. The approach can be described as simplified or naive, for three reasons: (i) position, orientation and size are sampled independently, which is to say that, for example, the size of a leaf may not affect its orientation; (ii) simple controls for phyllotaxy and clumping effects are yet to be implemented (although there is some control when generating the petioles); and (iii) the only effect leaves have on one another is that they are prevented from intersecting. We call this procedure the foliage and needles naive insertion algorithm, or the FaNNI algorithm in short.

#### 2.3.1. Overview of the procedure

**Figure 3.**
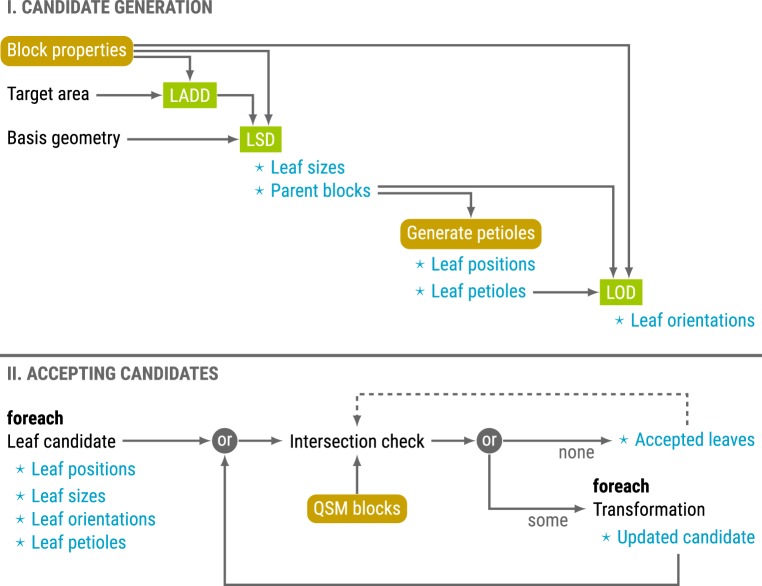
Process overview of the leaf generation process. Leaf distributions are drawn in rectangular boxes, and functions and properties related to the QSM in boxes with rounded corners. The main outputs are noted with a star. The two stages are presented on top of one another. (Online version in colour.)

The inputs of the algorithm are a collection of QSM blocks, leaf basis geometry, target leaf area to be distributed, and petiole and leaf parameter distributions. Details of the roles of the leaf basis geometry and the distributions are presented in §§2.3.2 and 2.3.3, respectively. The process can be viewed as two separate stages: (I) generating candidate leaves and (II) accepting candidates while preventing intersections. An overview of the process is provided in figure 3.

The first stage begins by distributing the available leaf area onto the blocks. The leaf area density distribution (LADD) determines the relative probability for a block with given parameters to have leaf area. After sampling the distribution with the block properties, each block has a target leaf area, or a leaf area budget, that will be divided into individual leaves by sampling the LSD.

For the leaf size determination the blocks are processed in random order. To match the target leaf area as closely as possible the cumulative area difference with respect to the target is updated after each leaf. While there is room in the current block, or the cumulative area budget, a new leaf is added to that block. The algorithm assumes that all the generated leaves have the same geometry, and thus we can sample a leaf length value which can be converted to area. After this step, the number of generated leaves and the block parent of each leaf are known.

Next, the locations of the leaves are determined by physically attaching them to their branches by the petioles. Because TLS measurements usually cannot capture petioles as they are too small to be detected reliably, all the petioles are generated: the petiole's starting point, orientation and length are determined by sampling appropriate parameter distributions given by the user. The end point of a petiole also determines the origin of the respective leaf. Although the exact petiole geometry is computed, they are considered insignificant compared with the blocks and the leaves, and thus they are excluded later from the intersection detection process.

The final property to sample is the leaf orientation. The LOD is used to determine the direction and the surface normal of each leaf. Once this is done, all leaves have a fixed position, orientation and scale, and their geometry can be computed by transforming the leaf basis geometry accordingly.

At this point it is possible, and even likely with a high leaf count, that some of the leaf candidates intersect one another, or the blocks, as they were generated independently. However, the goal is to produce a model without leaf intersections, and thus in the second stage the leaves are checked one by one for intersections before adding them to the list of accepted leaves.

If a leaf candidate intersects a block or an accepted leaf, it is possible to try to change the position, orientation and scale of the leaf and check whether the intersection was avoided. If it was, the leaf candidate is accepted; if not, the process can be repeated any number of times with a different transformation applied to the parameters. If, despite all the transformations, intersections cannot be avoided the candidate is discarded. An example of how intersection prevention can be implemented is described in §2.3.4. The leaf generation process stops when all the leaves have been processed, unless some other stopping condition has been given, such as a target leaf area of accepted leaves.

#### 2.3.2. Leaf model

The leaf model defines the basis geometry of an individual leaf. This geometry is the same for all the sampled leaves, but it is scaled, rotated and translated to receive the final leaf geometry, during the generation process. Thus all the generated leaves have the same shape but the size and orientation can vary. In the simplest case, the basis geometry can be a single triangle, allowing fast leaf cover generation due to simple intersection detections. For examples of basis geometries consisting of triangles, see §2.6. On the other hand, there is no upper limit for the complexity of the basis geometry, other than computational time requirements to ensure non-intersecting leaves. Thus, it is possible to represent more complicated shapes, e.g. a leaf with three-dimensional curvature, or a compound leaf with several leaflets, that do not have to lie on the same plane. However, to simplify the generation process, it is possible to use a simplified basis geometry while generating the leaves, which is then replaced with something more complex, as long as the change does not introduce additional intersections.

The origin of the leaf basis coordinate system is assumed to be the point where the petiole connects to the leaf. Leaf *direction* is the direction from the origin towards the tip of the leaf, and perpendicular to this lies the leaf *normal* that defines the direction to which (most of) the leaf area is facing. The length of the basis geometry, i.e. leaf length, is fixed at unity. Other dimensions are given with respect to that. During leaf parameter sampling only the leaf length is sampled as it determines the leaf area when the basis geometry is fixed. Note that it is not required to compute the exact geometry of the leaf candidates before the intersection prevention stage.

#### 2.3.3. Leaf and petiole parameter distributions

Leaf and petiole properties are controlled by multiple user-definable distributions which are sampled when leaves are generated. The properties fix the number of leaves, their position, size and orientation. In theory, these distributions are multidimensional as they may depend on any number of block properties, such as height from the ground, radius and orientation. They can also be formed as a weighted product or sum of one-dimensional marginal distributions. The purpose of each distribution is described below in the order they are sampled in the implementation.

##### 2.3.3.1. Leaf area density distribution

Total leaf area is one of the inputs of the algorithm, and leaf area density distribution defines how that area should be distributed to the blocks. Thus, the leaf area density distribution can allocate more leaf area towards the top of the tree and towards the tips of the branches. One could also prevent leaf area from being attached directly to stem blocks by using branch order information. Furthermore, the distribution produces a relative mapping of area on the blocks, allowing the distribution to assign any given total area of leaves to the structure model.

##### 2.3.3.2. Leaf size distribution

After a leaf area target has been assigned to each block, the LSD is used to sample leaf count and size, so that the target area is matched as closely as possible. This distribution determines the number of leaves to be generated *N*_init_. However, as no intersections between leaves or between blocks and leaves are tolerated, the final number of leaves may be smaller than initially generated if intersection cannot be avoided with transformations, i.e. *N*_final_ ≤ *N*_init_ holds.

##### 2.3.3.3. Petiole generation

After size distribution sampling, the number of leaves is known and it becomes possible to sample the petioles that connect the leaves to their block parents. Similarly to leaves, petiole parameters include the starting point, orientation and length of the petiole, which effectively also determine the starting points, or origins, of the leaves. It would be possible to model the petioles as three-dimensional objects, like small cylinders, but the implementation considers them only as line segments, and they are excluded from the intersection prevention step.

##### 2.3.3.4. Leaf orientation distribution

The final distribution controls the orientation of the leaves. This distribution controls the directions and normals of the leaves, and can be used to describe, for example, which parts of the tree are *erectophile* and which are *planophile*.

#### 2.3.4. Intersection prevention

Sampling the presented leaf and petiole parameter distributions results in a list of *N*_init_ candidate leaves. But because each sample is independent of the rest, the leaves may intersect with other leaves in the list, or blocks of the QSM. To avoid intersections, leaves are only accepted to the final collection of leaves if they do not intersect with other geometry.

The accepted leaves list is initialized as empty. One by one, the initial leaves are checked, so that they do not intersect with any of the blocks or the accepted leaves. To avoid a low acceptance rate, an intersecting leaf is not discarded instantly. Instead, a number of preselected user-defined transformations are applied to the leaf candidate, and intersection checking is repeated. A transformation may consist of any combination of scaling, rotation and translation, but they are applied in that order. Only if none of the preselected transformations prevent all the intersections, the candidate is discarded.

### 2.4. Leaf density model

Section 2.3 described an algorithm to generate exact leaf geometry by sampling certain distributions that depended on individual block parameters. However, in some cases it is not necessary to compute the exact geometry, but rather to view the leaves as an abstract density around the branches [19]. Such an approach saves computational resources as there is no need to compute and store a lot of geometry. This is especially relevant for computations with needles as their number often far exceeds the number of broadleaves for similar sized trees. This abstract approach without exact leaf realizations can be suitable for many applications, e.g. ray tracing operations in radiative transfer and gap fraction computations. However, exact geometry may be better suited for some applications, e.g. requiring realistic visualization, and it is also a more straight-forward way to study effects on a single broadleaf of needle scale.

**Figure 4.**
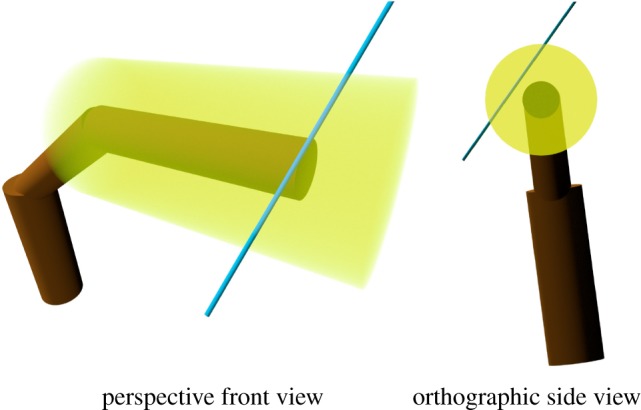
Two views of an example ray (blue) travelling through the leaf density cylinder (yellow) that is supported by one of the branch cylinders (brown).

The distributions defined earlier depended on block properties, which essentially means that each block defines a density, size and angle distribution around itself. In the case of a cylindrical QSM, this can be viewed as a *leaf density cylinder* around the block (figure 4). The radius (and length) of the leaf cylinder is defined by petiole length and LSDs. Let us next briefly justify the leaf cylinders as potentially useful and consider ray tracing with leaf cylinders as an example. One possible approach for ray tracing applications would be to determine an absorption rate for the leaf cylinder, which can depend on the distance from the cylinder axis, and where the rate can be stochastic (cf. the turbid medium analogy [4]). Branch cylinders can be viewed as infinitely dense, and thus hits occur at their surface. When enough of the energy of a simulated beam is absorbed, a hit occurs inside a leaf cylinder. If the application requires it, an incidence angle can be sampled from the orientation distribution stored in the respective block.

### 2.5. Inserting needles

Although this paper focuses on demonstrating broadleaf insertion, it is possible to use the algorithm with needles in different ways. The most obvious method is to use a tiny cylinder to represent a single needle and use that as a basis geometry. However, the computational requirements of the insertion would be enormous (but not impossible [20]), as they would be for any further application using the resulting model.

A less resource-consuming approach would be a modification of the leaf density cylinder approach described in §2.4. Rather than inserting needles at all, they could be viewed as a density distribution around the blocks (cf. [19]). Note that the distribution does not have to be uniform, and thus it can be used to account for needle phyllotaxy. Additional buds could also be introduced as density cylinders if the QSM does not contain the level of detail in terms of branching structure required by the user. Even though exact needle geometry is not generated, it is important to incorporate the needle phyllotaxy in any ray tracing operations inside needle density cylinders, as it is key in simulations including needles [21].

**Figure 5.**
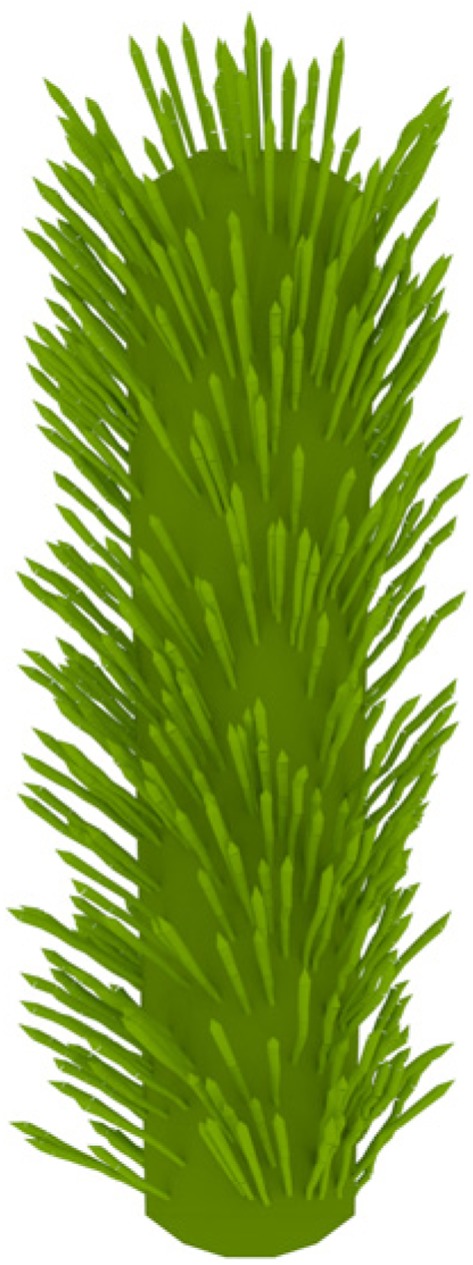
An example of a needle bud three-dimensional model without a strict phyllotaxy. (Online version in colour.)

A third option would be to use a needle bud as the basis geometry. An example of a needle bud suitable for visualization applications can be seen in figure 5. Even though the model is complex, it can be simplified to a cylinder during the intersection checking stage. The complex model can still be used for visualizations, or in further computations when required.

### 2.6. A Matlab implementation

The leaf insertion algorithm was implemented in Matlab [22]. The supporting classes and the main function of the implementation are presented below. Currently the implementation works with leaves, where the basis geometry is a collection of triangles, and cylindrical QSMs, but the structure of the implementation is modular, so that it is easy to extend to other types of leaves and blocks as necessary.

#### 2.6.1. Classes

The following classes were written to make the implementation as modular as possible. Especially, the LeafModel and QSMB abstract classes were designed to define interfaces for easy extendibility when using other structures than cylindrical QSMs, or triangle-based leaf models.

##### 2.6.1.1. LeafModel

The objects of this class have two main purposes in terms of the data they hold. First, they contain the leaf basis geometry, which is transformed to determine the geometry of the generated leaves. Second, they hold the parameters of the accepted leaves, i.e. leaf origin, scale, direction and normal. In terms of functionality the class is responsible for defining an intersection detection method for two leaves. There is also a method for converting the geometry of a leaf into a collection of triangles. The triangles method is required mainly when detecting intersections between a leaf and a block.^1^ There is also a method for adding a new, accepted leaf to the model.

**Figure 6.**
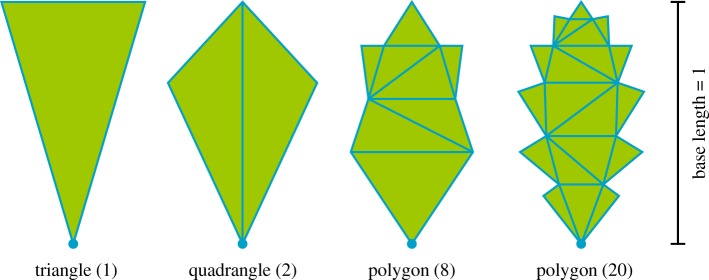
Triangular basis leaf geometries. The number of triangles is given in parentheses. The origin of the leaf is marked with a circle, and the length of a basis geometry always equals 1. (Online version in colour.)

LeafModel is an abstract class, used only for defining the required interface for subclasses rather than actually creating instances. This allows the class to be extended by creating subclasses, such as the implemented LeafModelTriangle class for leaf models, where the leaf basis geometry consists of vertices and triangular faces. This class already allows numerous leaf shapes, as seen in figure 6, but the user can extend the possibilities by implementing a subclass of LeafModel, e.g. for leaf geometry defined with Bézier curves, or other vertex–face-based geometries but with more optimized intersection detection than checking each triangle separately.

##### 2.6.1.2. QSMB

The class name is an acronym for quantitative structure model blocks (QSMBs), and it essentially acts as a container for QSMB information. The class is abstract and used to define an interface for its subclasses. The interface includes a method for reading block properties, such as position, orientation and branch order, and to detect intersection between blocks and triangles. Furthermore, a QSMB object is responsible for generating the petioles of the leaves using the block geometry. Finally, there is a method for converting the blocks of a QSM into a CubeVoxelization object, which is used to optimize intersection detection.

As an example subclass, the QSMBCylindrical was created to contain cylindrical QSM data. In this class, the block data consist of cylinder parameters for the geometry, and branching topology, such as branch order information. The user can extend the implementation to work on other types of structure models, by providing the appropriate subclass definition.

The QSMBCylindrical class also defines default uniform distributions for the petiole parameters. In this initial implementation, the petiole parameters are the following, with the lower and upper limits in parentheses: relative position along the cylinder axis (0, 1); relative position in the radial direction when connected to the end circle of the last cylinder in a branch (0, 1); rotation around the cylinder axis (−*π*, *π*); petiole elevation (−*π*/2, *π*/2); petiole azimuth (−*π*/2, *π*/2); and petiole length (2 cm, 5 cm).

##### 2.6.1.3. CubeVoxelization

An object of this class is a voxelization of a fixed three-dimensional space into cubical voxels with a fixed edge length. A CubeVoxelization object has a minimum and a maximum point and the space between them is divided into a finite number of cells. Object references can be stored in the cells to indicate that the objects occupy at least a part of that voxel. In the main function of the leaf insertion implementation, voxelizations are used to store and find candidate leaves and blocks, to perform more accurate intersection detection. Furthermore, the edge length of the voxelizations is set as the maximum leaf size produced by sampling the LSD function.

#### 2.6.2. Main function

qsm_fanni is the main function that receives the QSM as a QSMB object, an initialized LeafModel object that contains the leaf basis geometry, and total leaf area to be distributed. The leaf area parameter can have two components; one for the initial leaf area *A*_init_ to be generated, and one for the target leaf area *A*_target_ ≤ *A*_init_. This can be used to increase the probability that the target area is reached, even if some of the generated leaves are discarded due to unavoidable intersections.

There are also numerous optional inputs for the user to customize, such as the distribution functions and transformations during the intersection prevention step. However, default options are available for all the remaining parameters.

The main output of the function is a LeafModel object derived from the corresponding input, but it now contains the accepted leaves, petiole start points and a vector of parent block indices of each accepted leaf.

#### 2.6.3. Default leaf parameter distributions

The implementation contains default distribution functions for leaf parameter properties, and they are described below. At the moment these defaults are not designed to be biologically accurate, but rather just to provide an example of distributions. However, there are plans to improve the realism and usability of the default options in future versions, by offering the user a choice between common options, such as a spherical distribution for the leaf orientation.

##### 2.6.3.1. Leaf area density distribution

By default the available leaf area is distributed equally to all the last cylinders in the branches of the QSM. All other cylinders remain leafless.

##### 2.6.3.2. Leaf orientation distribution

The default LOD is such that most of the leaf area faces upwards, but there is some random variation. The LOD computes an initial leaf normal estimate as a cross product of the petiole direction and a side direction on a horizontal plane. If the initial direction differs by less than 20° from a reference direction (straight up in this case), then the final normal direction is the reference direction. Otherwise, the final normal is the initial direction rotated towards the reference direction by 20°.

##### 2.6.3.3. Leaf size distribution

The default LSD samples a leaf length value from a uniform distribution with given limits. That value is then scaled with a value based on the relative height of the parent block to ensure that leaves are a little bit larger at the top of the tree.

## 3. Results

### 3.1. Leaf geometry complexity test

**Figure 7.**
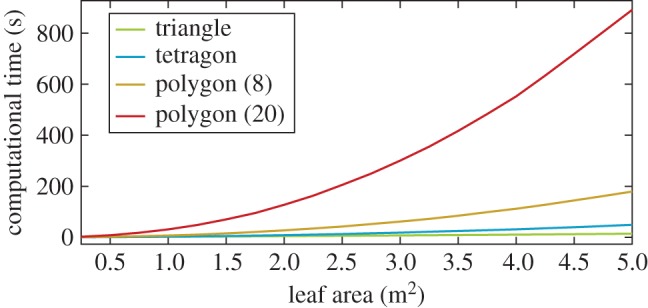
Computational time as a function of total generated leaf area for a single test cylindrical block. The values are averages over the 10 repeats.

The LeafModelTriangle class enables the use of leaf basis geometries with an arbitrary number of triangles. However, the detection of intersections between leaves requires that all those triangles are checked, which has an enormous effect on computational time. To study the effect of the number of triangles on the basis geometry, a single cylindrical block (length 1 m, radius 0.25 m) was fitted with an increasing total area of leaves. The area varied from 0.25 to 5 m^2^ for the four basis geometries in figure 6. The process was repeated 10 times for each leaf area–basis geometry pair. The average computational time results are shown in figure 7.

When using a single triangle, generating non-overlapping leaves was very fast even with the maximum leaf area, 5 m^2^, taking only 11 s on average. With the two-triangle quadrangle, the times increased 1.8-fold to 4.3-fold in comparison with the single triangle when moving from the lowest to the highest leaf area. For the polygon with eight triangles, the required time was 8.1-fold already at 1 m^2^ and 16-fold at the maximum. The respective multipliers for the 20-triangle polygon were 35.9 and a 79.7, which translate to 31 and 891 s, respectively.

### 3.2. Leaf area density distribution definitions

To demonstrate the leaf insertion algorithm, we defined the two following parametrized leaf area density distributions. While we tested other distributions and parametrizations, these two were chosen because of the low parameter count and overall simplicity.

**Figure 8.**
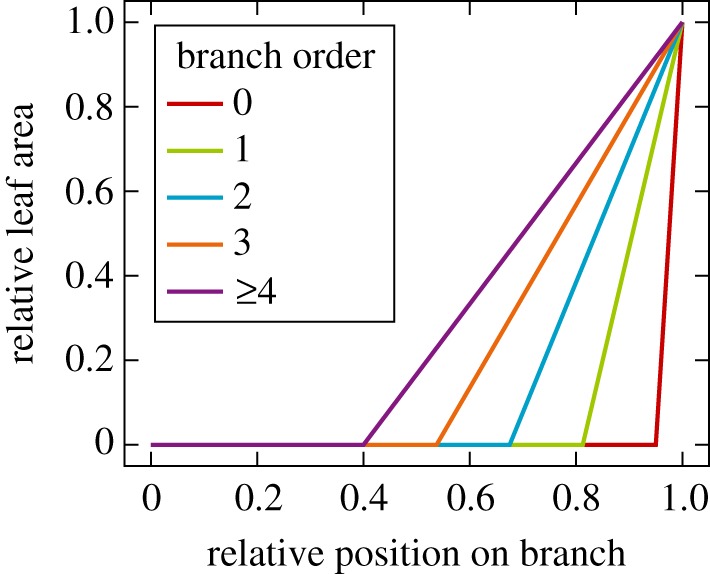
Piecewise linear polynomials defining the branch order-dependent LADD 2 scaling factor *y*_4_ = 0.4.

LADD 1 initialized the last 5% of each branch to have an equal portion of leaves, then scaling these proportions with a factor dependent on the relative height of the respective cylinder. The factor had a value of the parameter *y*_0_ at ground level and 1 at the top of the tree. Values in between were interpolated linearly.
LADD 2 had an additional parameter to define a cut-off point along a branch. The branch did not have any leaves before this point, which was dependent on the branch order. For the stem the cut-off was at 95%. For branch orders 4 and above, the cut-off was at *y*_4_, and for lower branch orders the cut-off was interpolated linearly. For cylinders after the cut-off point, the probability of leaves was interpolated linearly between 0 at the cut-off and 1 at the tip of the branch. Furthermore, the probabilities were scaled with a factor depending on the relative cylinder height as with LADD 1. The scaling factor *y*_4_ is visualized in figure 8 for a parameter value of 0.4.

**Figure 9.**
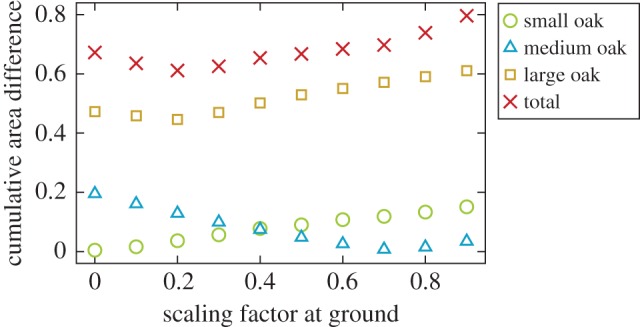
Cumulative area difference curves for the LADD 1 distribution as a function of the height scaling parameter. (Online version in colour.)

To find the optimal values for the parameters, we performed a simple grid search by varying the values of *y*_0_ and *y*_4_ in the closed intervals (0, 1) and (0, 0.9), respectively. For LADD 1, which only depends on the *y*_0_ parameter, the results are shown in figure 9; for LADD 2, the optimal parameter values are listed in table 3. Optimization was done on the cumulative area difference that was computed as the sum of unsigned leaf area differences in the vertical layers of the trees. The error was normalized with the measured total leaf area of the tree. The total error was computed as a sum over all the trees.

**Table 3.** Optimal parameter values for LADD 2 distribution. Parameter *y*_0_ controls the vertical distribution and parameter *y*_4_ the distribution along the branch length.

tree	*y*_0_	*y*_4_
small oak	0.1	0.7
medium oak	0.6	0.5
large oak	0.2	0.9
total	0.2	0.5

For LADD 1 the total optimal value was *y*_0_ = 0.2, which was close to those of the small and large oak trees. However, the optimal value of the medium oak tree was different at 0.7. For LADD 2 the total optimum values were *y*_0_ = 0.2 and *y*_4_ = 0.5, but there were differences in the optimal parameter values between the individual trees.

**Figure 10.**
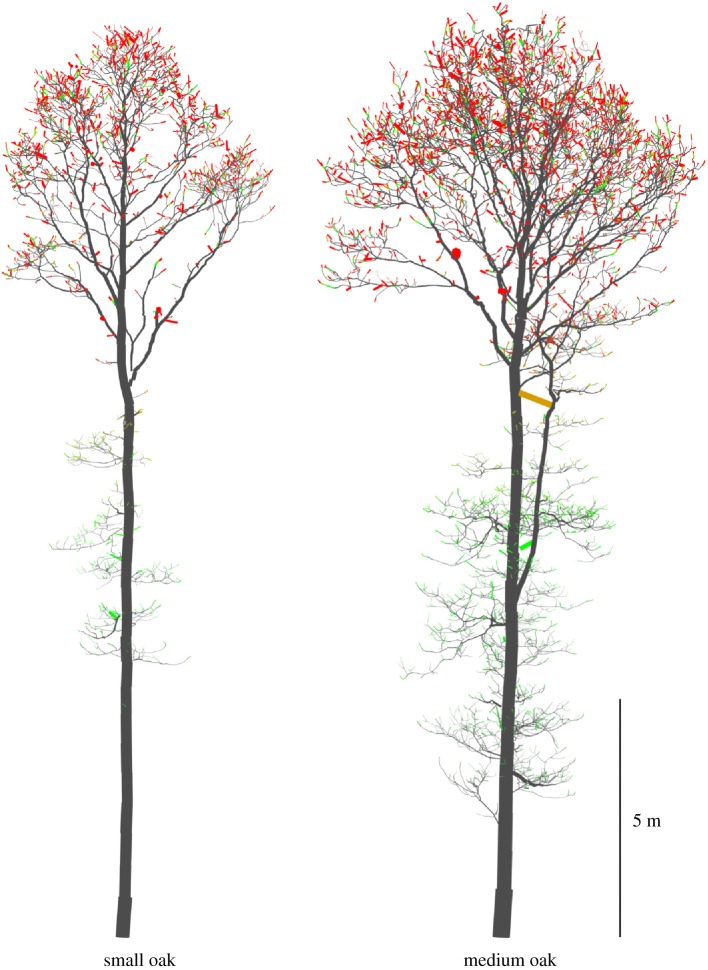
Example leaf area density distribution (LADD 2) for the small and medium oak trees as heat maps. As branch tips are small in size all cylinder radii have been scaled up to four times larger according their LADD value for a better visualization.

**Figure 11.**
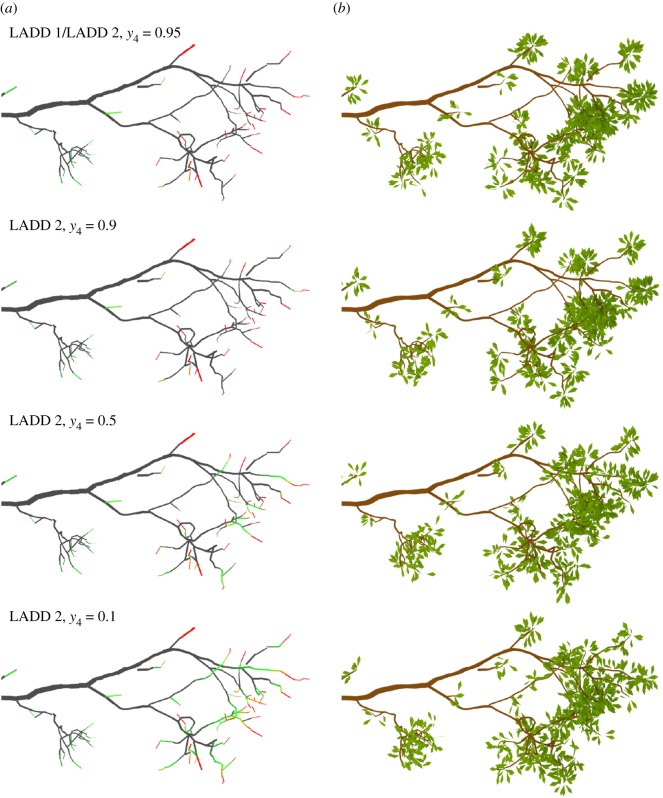
LADD examples on a single branch from the small oak tree. The distributions control how leaf area is distributed on the supporting branching structure. The parameter *y*_4_ controls the cut-off point along the branch length, starting from the branch base, before which there can be no leaves. (*a*) Distribution as a heat map; (*b*) sampled leaves based on the corresponding heat map.

Figure 10 visualizes the LADD 2 distribution with the optimal parameter values on the small and medium oak trees. Grey parts have no leaves, green parts have some, and red parts have a lot of leaves. Furthermore, figure 11 shows similar LADD heat maps and corresponding generated leaves. Note that in figure 11 LADD 1 is the same as LADD 2 with parameter value *y*_4_ = 0.95. Going from top to bottom the regions of high probability of leaves spread from the very tip towards the base of the branch. In the top two rows, the leaves are very concentrated at the tips, whereas in the latter two the leaves are more evenly spread along the high-order branches.

### 3.3. Leaf insertion test for oak trees

Each of the three oak trees was inserted with their measured leaf area (highlighted in table 1). The two LADDs described above with the optimal parameters were used, and all tree–LADD pairs were repeated 10 times. As we lacked reference data for the leaf orientation and LSDs, defaults from the Matlab implementation were used. To match the measured leaf sizes for each tree, the limits for the default uniform leaf length distribution were derived from the average leaf area measurements. The mean leaf length *l*_*i*_ for tree *i* was computed as follows:3.1
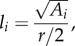
where *A*_*i*_ is the average leaf area for tree *i*, *r* ≈ 0.6 is the ratio between the width and length of the leaf basis geometry, which in this case was the quadrangle from figure 6 to keep the triangle count low. The leaf length limits were computed for each tree as *l* ± 1 cm.

The computations were done on a quad-core computer (Intel Core i7-6700 K 4 GHz, 32 Gb RAM). The computational mean times and standard deviations over the 10 repeats are listed in table 4. The average computational time per QSM block was between 20 and 40 ms for all the trees. Most of the computational time (95.3%) was spent on detecting intersections, which further supports using the simplest possible leaf basis geometry. The table also lists the average number of required block and leaf neighbour computations, the average number of performed transformations to avoid intersections, and the discarded leaf candidate percentage. The small oak tree had twice the leaf area per branch in comparison with the other two trees, which explains why there were twice as many neighbouring leaf computations and discarded leaves. The results suggest that it would be sufficient to sample 5–10% more leaves than the target leaf area to account for discarded leaves. The results show that the vast majority of leaf candidates are accepted without any transformation as the average number of tried configurations was between 1.0 and 1.5 for all the trees.

**Table 4.** Oak tree average leaf generation results. The properties are computational mean time, time standard deviation, average block and leaf neighbour counts, and average number of transformation configurations tried before accepting or discarding a leaf.

tree/LADD	time	time std	block neigh.	leaf neigh.	transforms	discard (%)
LADD 1						
small oak	6 min 12 s	7 s	13.1	32.8	1.4	7.3
medium oak	7 min 55 s	9 s	15.7	16.3	1.0	3.4
large oak	17 min 48 s	30 s	11.8	16.2	0.9	3.5
LADD 2						
small oak	6 min 32 s	4 s	13.6	33.9	1.4	7.8
medium oak	8 min 07 s	5 s	16.1	16.2	1.0	3.6
large oak	18 min 19 s	8 s	12.4	16.5	1.0	3.6

**Figure 12.**
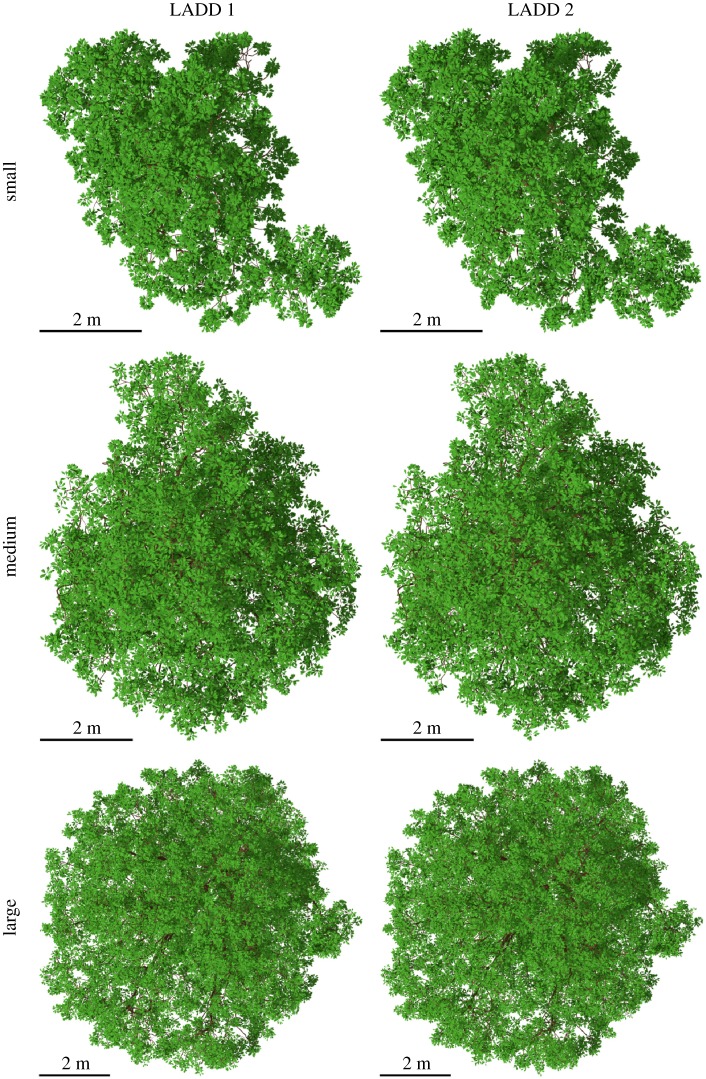
Top view of the three oaks with leaves generated with the two LADDs. (Online version in colour.)

**Figure 13.**
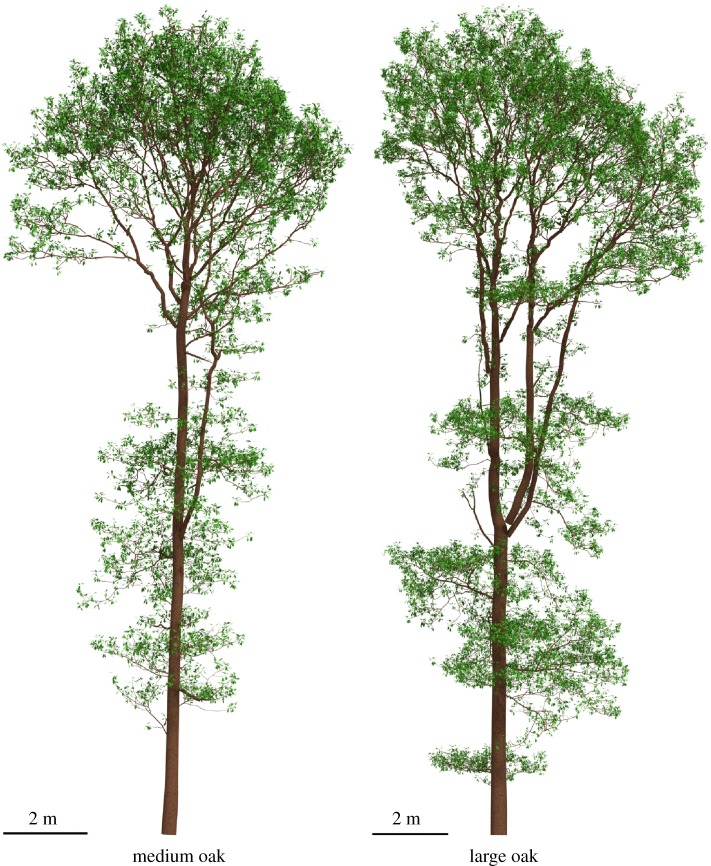
Side view of the medium and large oak with leaves generated with LADD 1. (Online version in colour.)

Figure 12 shows a top view of all the oak trees with leaves generated with both LADDs, and figure 13 shows a side view of the LADD 1-generated leaf covers for the medium and large oaks. The differences between the leaf covers generated with LADD 1 and LADD 2 are subtle, but notable. As the higher order branches have a lower cut-off point along the relative position on the branch, leaf cover is more even, making the gap fraction smaller on LADD 2 covers.

To compare the generated leaf distributions with the measured data, the leaves were placed in the same vertical bins listed in §2.1 according to their centre. The signed difference between generated and measured leaf count and area are listed in table 5. Negative values mean that the tree or layer should have had more leaves or leaf area; positive values are the opposite. Both LADDs were able to match the measured leaf area at the tree level because that was the stopping condition. The tree-level leaf counts are only between 500 and 3500 below the target values. Relative to the total leaf count the differences were 7.5%, 0.9% and 2.0% for the small, medium and large oaks, respectively.

**Table 5.** Difference between oak leaf count and leaf area in total and in vertical layers.

	LADD 1		LADD 2	
tree/layer	Δ count	Δ area (m^2^)	Δ count	Δ area (m^2^)
small oak	−3561	+0.0	−3581	+0.0
0.0–11.5 m	+1707	+5.7	+1002	+3.3
11.5–19.6 m	−5268	−5.7	−4583	−3.3
medium oak	−473	+0.0	−432	+0.0
0.0–9.0 m	−3339	−8.1	−2811	−6.0
9.0–19.9 m	+2866	+8.1	+2379	+6.0
large oak	−2275	−0.1	−2157	+0.0
0.0–8.0 m	+9507	+12.9	+10 748	+16.6
8.0–13.0 m	+2758	+13.1	+3254	+14.5
13.0–18.4 m	+15 634	+58.7	+15 883	+59.4
18.4–22.4 m	−30 174	−84.8	−32 040	−90.5

The layer-level differences were much higher, which suggests that the vertical distribution generated by the proposed LADDs did not match the measurements. With LADD 2 the top layer of the large oak was missing over 90 m^2^ of leaf area while the layer below that had an excess of about 60 m^2^. Results for the small oak were similar, which suggests lowering the *y*_0_ parameter. However, the opposite was true for the medium oak, which had about 6 m^2^ of extra leaf area in the upper layer.

## 4. Discussion

The above results presented two relatively simple LADD functions that used branch order, relative height and relative position along a branch to determine the portion of leaf area to be assigned to a block. However, the implementation allows for the user to write more complex LADD functions that make use of additional information, such as absolute height (whether the block is above the surrounding canopies) and absolute orientation (north or south side of the stem). Owing to limited reference data only the LADD was optimized. However, if detailed leaf angle or leaf size measurements are available, it is possible to optimize the respective distribution in a similar manner.

The LADD parameter optimization results and the conflicting layer difference results show that the presented LADDs are not able to capture the differences in the leaf area distributions of the three oak trees. Further studies should be carried out to assess whether the underlying leaf distributions differ between these three trees, or whether it is simply a matter of choosing a better LADD. It should also be noted that the manual leaf measurements were limited with only eight data points in total for the three trees, and, as such, more detailed and comprehensive measurements would be beneficial. Some of the leaf area difference can also be explained by uncertainties in estimating leaf area and count for the vertical layers, and by missing branches in the upper canopy in the QSMs.

The parameters of the two LADDs were optimized by using a grid search where exact leaf geometry was generated at each grid position. This made the optimization computationally intensive as 95% of the computational time was spent on intersection prevention, which forced a low parameter count. However, in retrospect it was unnecessary to generate leaf geometry, because as the results showed the discard rate was very low, which means that the LADD of the output was very close to the input. Thus, optimization according to, for example, vertical layers can be simplified to only include distributing the available leaf area onto the structure model and exclude both leaf size and orientation sampling and especially the computation of exact geometry.

Future research should also include testing the importance of the intersection prevention for various applications, i.e. whether possibly intersecting and non-intersecting leaves differ significantly in terms of required resources and produced level of detail. This way we would know whether it is sensible to perform the intersection prevention step, e.g. for simulations studying light use efficiency.

In this paper, the proposed method was only used to generate leaf covers according to user-given distributions. However, it would also be interesting to see whether this algorithm could be used to invert or approximate the real-leaf distributions of a given tree, with simple non-destructive and non-direct measurements. For example, it would be possible to test whether gap-fraction measurements and suitable parametrizations of the leaf distributions can be used to optimize the distribution parameters, to derive a mathematical or even a biological explanation for the real leaf distribution. With this method, it is possible to make such simulations and study this inverse problem. It should be noted that such inversion does not reconstruct exact leaf geometry but rather gives an approximation of their distributions. Such an approach could produce new understanding of what affects the distribution of leaves for a specific tree. Furthermore, it would allow the generation of leaf covers that follow the reconstructed distribution for the same tree or some other tree.

Currently the algorithm views each leaf independent from the others (apart from intersection prevention), which is one of the reasons for calling the algorithm naive. However, in most tree species leaves follow a certain phyllotaxy or the leaves are clumped together, e.g. their petioles originate near one another, or even from the very same spot [23]. We are planning to implement simple phyllotaxy controls in future versions of the FaNNI implementation. The level of clumping could be defined as a separate distribution that would be used to sample the size of a clump and variation in petiole and leaf parameters for the leaves within the clump.

In nature, leaves are often connected to branches that are small in diameter. Because of the limitations of the TLS technology, such branches are often poorly sampled in the resulting point clouds. Therefore, they can be excluded from the reconstructed QSM also, which means that, when leaves are inserted, they are connected to branches that are too large. To counter this shortcoming, it is possible to perform a pre-processing step that inserts small branches into the structure model, which will be given a high probability of leaves when defining the LADD function.

Although the implementation enables the use of leaf basis geometries consisting of any number of triangles, the results show that additional complexity multiplies the expected computational time by large factors. However, if detailed leaf geometry is required for later computations, it is possible to use a simplified stand-in basis geometry that encapsulates the complex shape to prevent overlapping during generation and replace the geometry afterwards. Such a procedure could even be built in to an extension of the LeafModel class.

## 5. Conclusion

We have presented an algorithm to generate non-intersecting leaves to a QSM that follow user-defined position, size and orientation distributions. A Matlab implementation of the algorithm was also presented. Currently, the implementation allows the use of any leaf shape consisting of an arbitrary number of triangles.

In order to present leaf property distributions in a compact yet versatile format, we propose a scheme where a QSM is divided into blocks that determine, and can be used to contain, property information for leaves that are to be connected to it. This means that we can assign the available leaf area, leaf size and orientation parameters to the blocks of a QSM even without generating leaves. Then we can do one of the following.

— Visualize the property distributions by colouring the blocks according to their respective property values as seen in the case of leaf area density distributions, e.g. in figures 10 and 11.
— Sample the user-defined distribution with the parameter values and generate exact leaf geometry as was done in §3.
— View the leaves as a probability distribution around the QSM blocks, and rather than computing exact leaf geometry do computations by determining the probability of a hit and the incidence angle when a beam enters the vicinity of a block.

Although any triangle-based geometry is possible for the leaves, a simple test of adding an increasing area of leaves to a single cylindrical block showed that complex leaf shapes can drastically increase the computational time, at least with the current implementation. Thus, the leaf basis geometry should be kept as simple as possible, or optimization is required for intersection detection.

To demonstrate leaf generation, we presented two different LADDs and applied them to three oak trees trying to match field measured leaf count and areas. The measurements were done with two--four vertical bins per tree, and the average leaf area was also recorded for each tree. Simple uniform LSD (with some scaling based on height) and planophile orientation distribution were used, while the main focus was on optimizing the LADDs. The two suggested LADDs were able to match leaf area and count per tree, but the vertical distribution of leaves had major errors despite the optimization. Further research is required to understand the cause of the leaf area differences.

A further goal is to use the leaf-augmented QSM (L-QSM) to incorporate a number of biological principles such as the availability of resources (mass and energy exchanges between vegetation and atmosphere, and phyllotaxy) to construct as many self-consistent tree models as possible. One can include stochastic variations in the same sense as in the creation of four-dimensional QSMs [24], extending that scheme to fully functional trees. This approach would enable a large number of applications to verify and refine assumed biological postulates of theoretical models, and then use the resulting full-scale three- and four-dimensional models for predictions and the modelling of ecological systems at various size and complexity scales, including large-scale statistical (allometric) estimates.

## Endnote

1 Otherwise you would have to write a separate intersection detection function for each leaf and block type pair.

## Data accessibility

The Matlab implementation of the FaNNI algorithm is available on GitHub (https://github.com/InverseTampere/qsm-fanni-matlab). The three oak tree QSMs are available from Eric Casella (Eric.Casella@forestry.gsi.gov.uk) upon request.
